# Supplementary material for: End Sequence Analysis Toolkit (ESAT) expands the extractable information from single-cell RNA-seq data
Source: Genome Res. 2016 Oct;26(10):1397–410. doi: 10.1101/gr.207902.116 (PMC5052061; doi:10.1101/gr.207902.116)
Supplement: Supplemental Material [file supp_gr.207902.116_Supp_Materials.tar.gz › ESAT-esat_v0.1/doc/html/dir_f319a9351526bf9df2b388f7d2ede6fa.html]

ESAT: java/broad/pda/seq/rap Directory Reference


ESAT


- Main Page
- Related Pages
- Packages
- Classes
- Files

All Classes Namespaces Files Functions Variables Enumerator Pages

- java
- broad
- pda
- seq
- rap

rap Directory Reference

Directory dependency graph for rap:

|  |  |
| --- | --- |
| Directories | |
| directory | rna |
|  | |

|  |  |
| --- | --- |
| Files | |
| file | BuildRatioNullDistribution.java |
|  | |
| file | CalculateCoverageStats.java |
|  | |
| file | CollectAnnotationEnrichments.java |
|  | |
| file | CountProbes.java |
|  | |
| file | CountReads.java |
|  | |
| file | DistanceToBED.java |
|  | |
| file | GCContent.java |
|  | |
| file | GenomeCommandLineProgram.java |
|  | |
| file | GenomeScoringProgram.java |
|  | |
| file | PlotAggregateRegions.java |
|  | |
| file | RAPPeakCaller.java |
|  | |
| file | RatioPermutationPeakCaller.java |
|  | |
| file | ShuffleBAM.java |
|  | |
| file | ShuffleBED.java |
|  | |
| file | SkellamKLPeakCaller.java |
|  | |
| file | SkellamScore.java |
|  | |
| file | SlideAndCalculate.java |
|  | |
| file | SlideAndCount.java |
|  | |
| file | UnmarkDuplicates.java |
|  | |


---

Generated on Fri Aug 15 2014 15:11:16 for ESAT by  

 1.8.7
